# Retrorsine impairs liver regeneration by inducing progenitor cell-senescence *via* ROS after partial hepatectomy

**DOI:** 10.1080/07853890.2026.2635821

**Published:** 2026-03-03

**Authors:** Yan Cui, Li Li, Yu He, Shan Shan, Lin Liu, Jiangbo Ren, Hui Wang, Miaoran Yang, Xinyan Zhao, Jidong Jia, Ping Wang

**Affiliations:** a Liver Research Center, Beijing Friendship Hospital, Capital Medical University, Beijing, China; b Institutional Office, Beijing Friendship Hospital, Capital Medical University, Beijing, China; c State Key Laboratory of Digestive Health and National Clinical Research Center for Digestive Diseases, Beijing, China

**Keywords:** Partial hepatectomy, retrorsine, hepatic progenitor cells, senescence

## Abstract

**Background:**

Hepatic progenitor cells (HPCs) provide an alternative regenerative pathway when hepatocytes are in a senescent state or after chronic liver injury. Yet, their contribution to liver regeneration after partial hepatectomy (PH) remains controversial. The aim of this study is to reveal the regeneration contribution of HPCs after PH when hepatocyte proliferation is totally suppressed.

**Methods:**

Retrorsine (RTS) was administered to suppress hepatocyte proliferation in C57BL/6J mice after PH. The regeneration contribution of HPCs was assessed using HPC-specific lineage tracing mice (Sox9^Cre-ER^Rosa^tdTomato^) after RTS/PH. The effects of RTS on HPCs were analyzed by HPC-enriched organoids *in vitro*.

**Results:**

RTS 0–70 mg/kg dose-dependently reduced the PCNA^+^ hepatocyte ratio on day 2 post-PH with hepatocyte proliferation completely suppressed by 70 mg/kg RTS. Yet, the proportion of PCNA^+^ HPCs did not increase in these 70 mg/kg RTS/PH mice, suggesting that HPCs were not activated when RTS completely inhibited hepatocyte proliferation. By day 14 post-PH, the 70 mg/kg RTS/PH mice exhibited reduced liver-to-body weight ratios compared to control mice, indicating regeneration failureafter PH. Simultaneously, the tdTomato-positive regenerative foci derived from labeled HPCs were not significantly different from those of the controls, indicating a minimal regeneration contribution from HPCs. RTS increased intracellular ROS levels and β-galactosidase activity of HPC-enriched liver oranoids *in vitro*, and phospho-H2A.X positive HPCs could be found in 70 mg/kg RTS/PH mouse liver, suggesting RTS-induced HPC senescence.

**Conclusion:**

When hepatocyte proliferation was completely suppressed by RTS, HPCs contributed minimally to liver regeneration owing to cellular senescence, resulting in regeneration failure.

## KEY MESSAGES

- HPCs contribute minimally to liver regeneration when hepatocyte proliferation is completely arrested by RTS, leading to liver recovery failure.
- RTS targets HPCs in the liver, in addition to hepatocytes and sinusoidal endothelial cells, and induces HPC senescence *via* ROS generation.
- RTS-induced regeneration failure was accompanied by hepatocyte hypertrophy and CD11b^+^Ly6G^+^ MDSC infiltration.

## Introduction

Liver possesses extensive regenerative capacity to restore lost parenchyma and regain liver weight after liver resection [1,2]. In rodents, liver mass typically regenerates within seven days and fully recovers within three weeks after two-thirds partial hepatectomy (PH), primarily through hepatocyte duplication [3,4]. Notably, mouse lineage-tracing studies have demonstrated a minimal contribution of hepatic progenitor cells (HPCs) to hepatocyte regeneration after partial hepatectomy (PH) [5,6]. However, when hepatocyte proliferation is pharmacologically inhibited using agents such as 2-acetylaminofluorene (AAF), which induces DNA adducts and cell-cycle arrest, the reported outcomes for HPC-driven regeneration are inconsistent. Some studies in AAF/PH models found that HPCs (oval cells) differentiate into hepatocytes [7,8], while others observed defective liver mass recovery, with HPCs expanding but failing to differentiate and rarely forming mature hepatocytes [9,10]. Given that progenitor cell differentiation requires complete suppression of hepatocyte replication, often coupled with senescence induction [11], this discrepancy in HPC differentiation within the AAF/PH model may be due to incomplete suppression of hepatocyte proliferation. Therefore, the contribution of HPCs to liver regeneration in case of suppressed hepatocyte proliferation after PH still needs further exploration.

AAF fails to effectively activate mouse HPCs [11] owing to metabolic differences, specifically the lack of a sulfotransferase required to block mouse hepatocyte proliferation [12,13]; therefore, it is necessary to suppress hepatocyte proliferation using other reagents for mouse lineage-tracing studies. Retrorsine (RTS), a natural pyrrolizidine alkaloid derived from *Compositae Senecio*, is known for its potent liver toxicity *via* the induction of hepatocyte damage in mice, rats and humans [14–16]. Fischer 344 rats deficient in dipeptidyl peptidase IV (DPPIV), pretreated with RTS and subjected to PH, demonstrated that liver mass restoration was mediated by a cell population resembling small hepatocyte-like cells rather than mature hepatocytes or oval cells [17,18]. This suggests that the contribution of HPCs is limited when small hepatocytes are involved in regeneration; however, their contribution when hepatocyte proliferation is completely suppressed after PH remains unclear.

Therefore, this study optimized the RTS dosage to suppress hepatocyte proliferation and used Sox9^ER-Cre^Rosa^Tdtomato^ HPC-specific lineage-tracing mice to assess the contribution of HPCs to PH. We found that 70 mg/kg RTS completely arrested hepatocyte proliferation, induced hypertrophy, and enhanced senescence. Concurrently, the liver-to-body weight ratio failed to recover to normal levels by day 14 post-PH, indicating a regeneration failure. No significant HPC expansion or minimal HPC-derived hepatocytes were observed, suggesting minimal regeneration from HPCs. *In vitro* analyses demonstrated that RTS induced senescence of HPC-enriched liver organoids by enhancing intracellular ROS levels. Thereby, RTS simultaneously impaired hepatocyte proliferation and HPC regeneration, thus resulting in liver regeneration failure.

## Material and methods

### RTS/PH mice

Thirty-two 8-week-old male C57BL/6J mice (Beijing HFK Bioscience Co., Beijing, China) were randomized into seven cages (≤ 5 mice/cage) and maintained under specific pathogen-free (SPF) conditions at the Experimental Animal Department of Beijing Friendship Hospital. RTS (10 mg, Yuan Ye Biol, Shanghai, China) was dissolved in distilled water (0.5 mL) by titrating to pH 2.5 with 1 N HCl. The solution was neutralized to pH 7.0 with 1 N NaOH, followed by the addition of NaCl to obtain a working solution with a final concentration of 10 mg/mL RTS and 150 mmol/L NaCl, which was used immediately [17]. After one week of acclimation, the mice received intraperitoneal (i.p.) injections of saline (*n* = 10, 5 for controls, the other 5 for 0 mg/kg RTS), 35 mg/kg RTS (*n* = 5), 70 mg/kg RTS (*n* = 7), or 105 mg/kg RTS (*n* = 10). Based on the pre-experimental data indicating RTS toxicity, the group sizes were increased by assigning two additional mice to the 70 mg/kg dose group and five to the 105 mg/kg dose group to guarantee an adequate sample size for final analysis. The mice receiving 105 mg/kg RTS died within 2 days. The remaining mice received a second injection of saline or RTS one week later. Subsequently, the saline (0 mg/kg RTS)- (*n* = 5), 35 mg/kg RTS- (*n* = 5), and 70 mg/kg RTS-treated (*n* = 7) mice were anesthetized (i.p. pentobarbital sodium, 40 mg/kg), and left lateral and right medial lobes (∼50% parenchymal) were ligated respectively and then resected, leaving the gallbladder intact, with minimal blood loss during the hepatectomy [19]. The remaining five saline-treated mice served as controls. Two mice in the 70 mg/kg RTS group died within 24 h post-PH, possibly due to RTS toxicity. The surviving mice were maintained under SPF conditions for two days after resection.

### HPC-lineage tracing mice

Sox9^Cre-ER^Rosa^tdTomato^ mice were generated by crossing Sox9^Cre-ER^ mice (Shanghai Model Organisms, Shanghai, China) with Rosa^tdTomato^ mice (Shanghai Model Organisms) with a C57B/6J background [20] and were maintained under SPF conditions. Twenty-two 6-week-old Sox9^Cre-ER^Rosa^tdTomato^ male mice were randomized into 5 cages (≤ 5 mice/cage) and Sox9^+^ cells were labeled with 8 mg/kg B.W. tamoxifen (dissolved in corn oil) by oral gavage. After a 2-week washout period, the mice received i.p. injections of saline (0 mg/kg RTS (*n* = 10), 35 mg/kg RTS (*n* = 5), or 70 mg/kg RTS (*n* = 7) twice after a one-week interval. Of them, 17 labeled mice underwent 50% PH (0 mg/kg RTS, *n* = 5; 35 mg/kg RTS, *n* = 5; 70 mg/kg RTS, *n* = 7) under anesthesia. The remaining five saline-treated mice served as controls (*n* = 5). Two mice in the 70 mg/kg RTS group died within 24 h post-PH, and the surviving mice were maintained under SPF conditions for 14 days post-resection.

### Sample collection

At the study endpoint, the mice were weighed, anesthetized (i.p. pentobarbital sodium, 40 mg/kg), and euthanized by cervical dislocation. The livers were perfused with saline *via* the ventriculus to remove blood cells and then excised for weighing. Tissues were processed for histology (H&E, immunofluorescence for PCNA/HNF4α, PCNA/Sox9, Tdtomato, HNF4α/β-catenin, HNF4α/p21, TUNEL, phospho-H2A.X/Sox9) and non-parenchymal cell (NPC) isolation. The NPCs were assessed by flow cytometry for proportions of CD45^+^CD3^－^ cells, CD11b^+^Ly6G^+^ cells (myeloid-derived suppressor cells, MDSCs), and Sox9^+^ cells. As an exploratory study, the sample sizes were determined based on our preliminary experiments to adhere to the ethical principle of reduction and achieve reliable scientific objectives. Data from four mice that died after resection in the 70 mg/kg RTS/PH group were excluded from the final analyses. Potential confounders such as treatment order, measurements, and animal/cage locations were not controlled. The researchers and data analysts were not blinded to the group allocation at different stages of the experiments. The animal experiments and procedures were approved by the Animal Care and Use Committee of Beijing Friendship Hospital, Capital Medical University. Title of the approved project: The effects of interrupting GITRL expression in HPCs on mouse liver regeneration and liver fibrosis. Approval number: 19–2021. Date of approval: 12/26/2019.

### Immunofluorescence staining

Standard immunofluorescence staining was performed as described previously [21]. Tissue sections were incubated with primary antibodies: mouse anti-PCNA (1:200, Cell Signaling #2586); rabbit anti-hepatocyte nuclear factor (HNF) 4α (1:200, Cell Signaling #3113) or mouse anti-HNF4α (1:200, Abcam ab41898); rabbit anti-Sox9 (1:200, Cell Signaling #82630); goat anti-tdTomato (1:400, Biorbyt orb182397), rabbit anti-p21 (1:200, ProteinTech 10355-1-AP), rabbit anti-β-Catenin (1:200, ProteinTech 51067-2AP), mouse anti-phospho-Histone H2A.X (Ser139, 1:200, Cell Signaling #80312). Subsequently, the sections were incubated with the following secondary antibodies: Alexa 488 (1:600; Molecular Probes A-21202 or A-21206) and/or Alexa 555 (1:600; Molecular Probes A-21432, A-31570, or A-31572). The antibodies used are listed in Supplementary Table 1. Sections were visualized using a Nikon Ts2 FL Microscope and scanned using a Panoramic MIDI Digital Slide Scanner (3DHISTECH).

### Flow cytometry analysis

Liver NPCs were isolated by collagenase digestion and Percoll density separation as described previously [20]. The fixed and permeabilized cells were stained with PE-anti-CD45 (BioLegend 147712) and Alexa Fluor 647-anti-Sox9 (BD Biosciences 565493) antibodies for HPC analysis. The cells were stained with BV421-anti-CD45 (BioLegend 103134) and PE-Cy7-anti-CD3 (BioLegend 100220) antibodies for immune cell analyses. The cells were stained with BV421-anti-CD45 (Biolegend 103134), PE-Cy7-anti-CD11b (Biolegend 101216), and PerCP/Cy5.5-anti-Ly6G (Biolegend 127616) antibodies for CD45^+^CD3^－^ subset analyses. Data were acquired on an LSRFortessa flow cytometer (BD Biosciences) and analyzed using the FlowJo software (Treestar).

### Cell culture and RTS treatment

The mouse hepatocyte line H2.35 (Bo Ao Kai Mei Tech, Beijing, China) was cultured in DMEM/F12 medium supplemented with 10% fetal bovine serum and 1% antibiotics. Mouse liver organoids were derived from male C57BL/6J mice and cultured as described previously [20]. H2.35 cells or liver organoids were treated with 0, 30, 60, or 90 µM RTS for 48 h.

### qRT-PCR

Total RNA was extracted from mouse samples using the TRIzol reagent (Invitrogen, Carlsbad,CA, USA) according to the manufacturer’s instructions. RNA reverse transcription, polymerase chain reaction, and data analysis were performed as described previously [22]. The primers used are listed in Supplementary Table 2. GAPDH was used as an endogenous control.

### β-galactosidase (β-gal) staining

β-gal activity was detected using the Senescence β-Galactosidase Staining Kit (Cell Signaling #9860). Briefly, the control or RTS-treated organoids were fixed with 1× Fixative Solution for 15 min at room temperature. After rinsing twice with PBS, fixed organoids were incubated with the β-gal staining solution (pH 6.0) overnight at 37 °C in a CO_2_-free, dry incubator. Blue color development, indicating β-gal activity, was observed under a Nikon Ts2 FL microscope.

### Intracellular ROS measurement

Reactive oxygen species (ROS) levels were measured using a Reactive Oxygen Species Assay Kit (Applygen C1300, Beijing) according to the manufacturer’s instructions. Briefly, control or RTS-treated organoids were washed twice with PBS and incubated with 10 µM DCFH-DA in serum free DMEM/F12 medium for 1 h at 37 °C in 5% CO_2_ incubator. After washing with PBS, the ROS-associated green fluorescence was visualized using a Nikon Ts2 FL Microscope.

### Statistical analysis

Data are presented as mean ± SD. If the data variances were similar between groups, they were analysed for significance using an unpaired *t*-test with a two-tailed *p*-value, while if the data variances were not similar between groups, they were analysed for significance using an unpaired *t*-test with Welch’s correction *via* two-tailed *p*-values with GraphPad Prism 6 software (GraphPad, La Jolla, CA, USA). *p* < 0.05 was considered statistically significant.

## Results

### RTS (70 mg/kg) completely suppressed hepatocyte proliferation by day 2 post-PH

**Figure 1.**
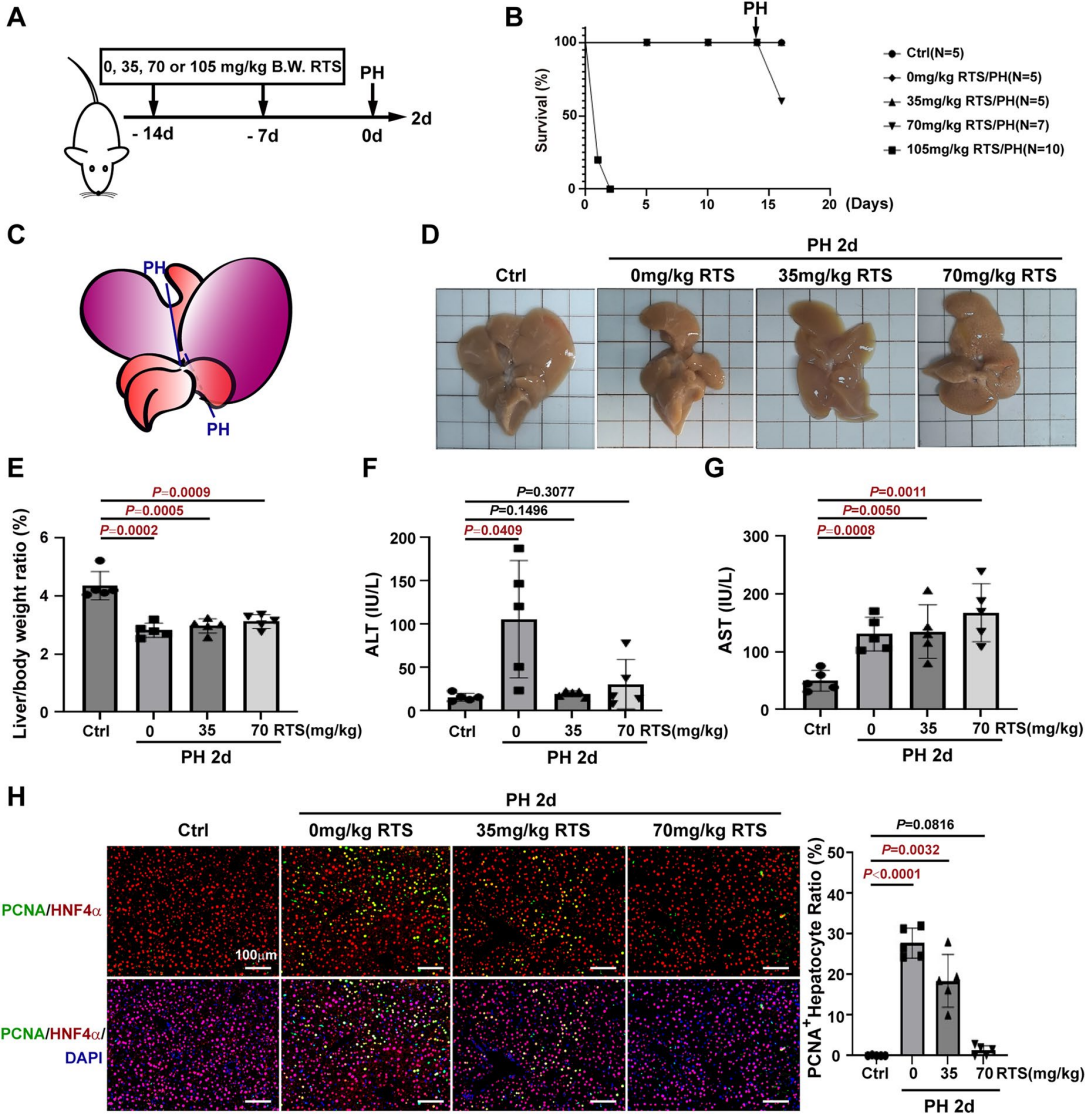
**RTS suppressed hepatocyte proliferation by day 2 post-PH in a dose-dependent manner.** (A) Experimental design to determine the effects of different dose of RTS on C57BL/6J mice by day 2 post-PH. (B) The survival curve after different dose of RTS (0, 35, 70, and 105 mg/kg) treatment covered the period extending from the initial RTS treatment to 2 days after PH. (C) The left lateral lobe and the right medial lobe were surgically removed for hepatectomy. (D) The representative macroscopic view of livers from controls, 0 mg/kg, 35 mg/kg, or 70 mg/kg RTS/PH mice by day 2 post-PH. (E) By day 2 post-PH, the liver-to-body weight ratios were significantly lower in 0 mg/kg, 35 mg/kg, or 70 mg/kg RTS/PH mice compared to controls. (F) Seum ALT levels in the control, 0 mg/kg, 35 mg/kg, or 70 mg/kg RTS/PH mice. (G) Seum AST levels in the control, 0 mg/kg, 35 mg/kg, or 70 mg/kg RTS/PH mice. (H) Double immunostaining for PCNA and HNF4α revealed RTS completely suppressed hepatocyte proliferation in 70 mg/kg RTS/PH mice by day 2 post-PH.

The mice received different doses of RTS (35, 70, or 105 mg/kg B.W.) once a week, starting 14 days before PH to inhibit hepatocyte proliferation (Figure 1A). All mice that received two injections of 35 or 70 mg/kg RTS survived; however, mice that received one injection of 105 mg/kg RTS died within 2 days (Figure 1B). Additionally, resection of the left lateral and right medial lobes (Figure 1C) resulted in two deaths within 24 h in the 70 mg/kg RTS/PH group, whereas no deaths occurred in the 0 or 35 mg/kg RTS groups (Figure 1B). The remnant liver lobes appeared enlarged by day 2 post-PH compared with the control lobes (Figure 1D). The liver-to-body weight ratios in the 0 mg/kg RTS/PH (2.821% ± 0.2463%, *n* = 5), 35 mg/kg RTS/PH (2.972% ± 0.2460%, *n* = 5), and 70 mg/kg RTS/PH (3.121% ± 0.2397%, *n* = 5) groups were significantly lower than those in the control group (4.361% ± 0.4877%, *n* = 5) (Figure 1E). The ALT levels were increased in 0 mg/kg RTS/PH group (Figure 1F), while AST levels were markedly increased in 0 mg/kg RTS/PH (130.6 ± 29.28, *n* = 5), 35 mg/kg RTS/PH (135.0% ± 46.37%, *n* = 5) and 70 mg/kg RTS/PH (167.8 ± 50.23, *n* = 5) groups than that in the control group (49.95 ± 17.81, *n* = 5) (Figure 1G). Immunofluorescence staining for PCNA and HNF4α revealed that proliferating hepatocytes were significantly higher in the 0 mg/kg RTS/PH (27.78% ± 3.660%, *n* = 5) and 35 mg/kg RTS/PH (18.49% ± 6.500%, *n* = 5) groups than that in the control group (0.04343% ± 0.09711%, *n* = 5) (Figure 1H). In contrast, proliferating hepatocytes in 70 mg/kg RTS/PH group (1.280% ± 1.196%, *n* = 5) were similar to that in the control group (Figure 1H), confirming that 70 mg/kg RTS suppressed hepatocyte proliferation after PH.

### HPCs were not activated when hepatocyte proliferation was suppressed by RTS on day 2 post-PH

**Figure 2.**
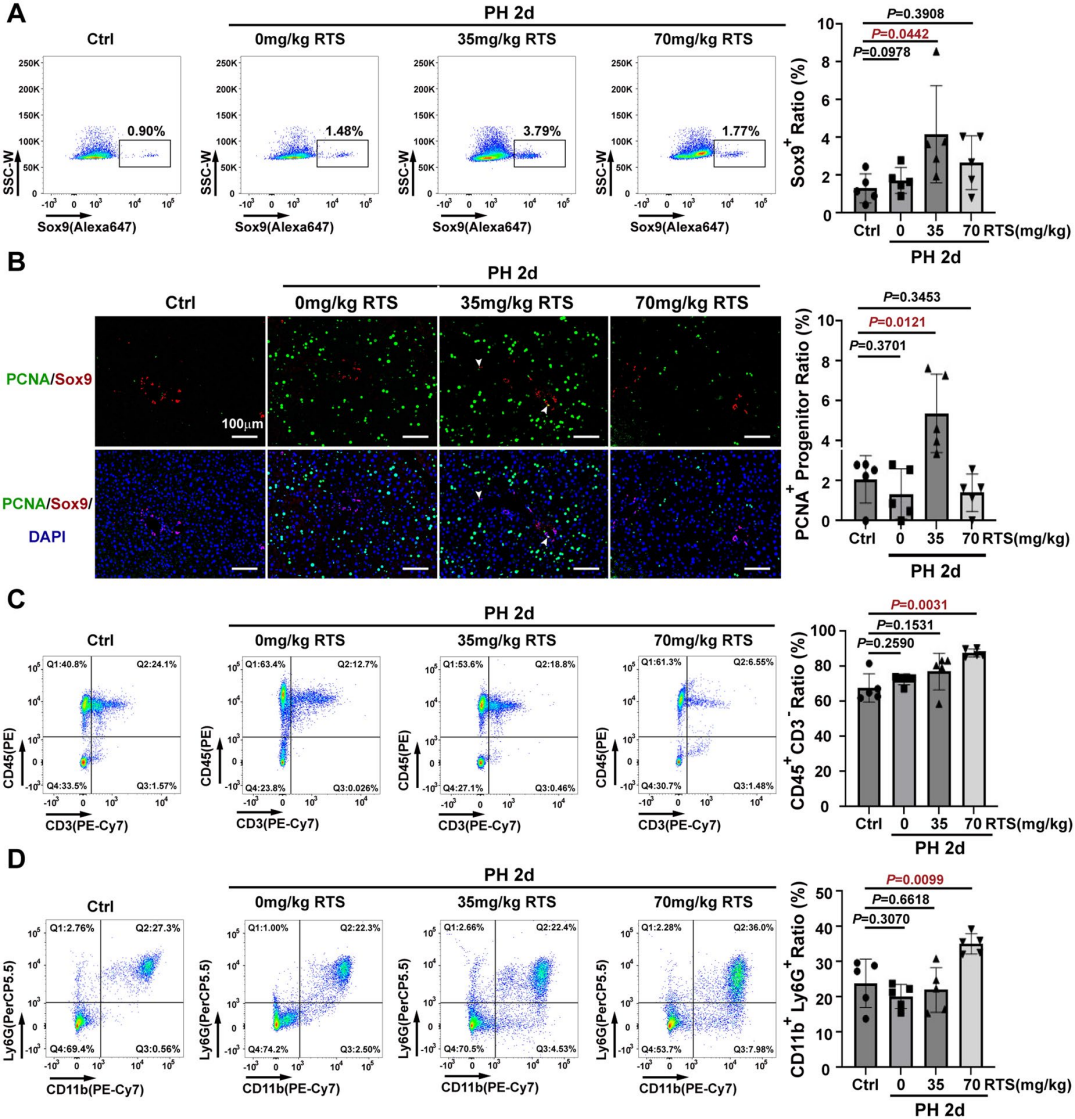
**No significant HPC expansion in RTS/PH mice when hepatocyte proliferation was completely suppressed by day 2 post-PH.** (A) The proportion of Sox9^+^ HPCs were not significant changed in 70 mg/kg RTS/PH mice compared to controls by day 2 post-PH. (B) PCNA^+^ HPCs were not markedly changed in 70 mg/kg RTS/PH mice compared to controls by day 2 post-PH. (C) The proportion of CD45^+^CD3^–^ cells among CD45^+^ cells were higher in 70 mg/kg RTS/PH mice compared to controls by day 2 post-PH. (D) The proportion of CD11b^+^Ly6G^+^ MDSCs among CD45^+^ cells was higher in 70 mg/kg RTS/PH mice compared to controls by day 2 post-PH.

In the analysis of progenitor cells, multicolor flow cytometry showed that the proportion of Sox9^+^ HPCs (among the NPCs) was significantly higher in the 35 mg/kg RTS/PH group (4.170% ± 2.566%, *n* = 5) than that in the control group (1.314% ± 0.7669%, *n* = 5), but not significantly different from that in the 0 mg/kg RTS/PH (1.732% ± 0.6880%, *n* = 5) or 70 mg/kg RTS/PH (2.666% ± 1.420%, *n* = 5) groups (Figure 2A). Consistently, double immunofluorescence staining for PCNA and Sox9 revealed that the number of proliferating HPCs was significantly higher in the 35 mg/kg RTS/PH group (5.381% ± 1.956%, *n* = 5) than that in the control group (2.079% ± 1.186%, *n* = 5), but was not significantly different from that in the 0 mg/kg RTS/PH (1.343% ± 1.262%, *n* = 5) or 70 mg/kg RTS/PH (1.401% ± 0.9354%, *n* = 5) groups (Figure 2B). These data suggested that HPC activation did not occur when hepatocyte proliferation was completely suppressed by RTS following PH.

Therefore, multicolor flow cytometry was performed to identify the immune cells in the liver by inhibiting hepatocyte proliferation and blocking HPC activation. The results showed the proportion of CD45^+^CD3^－^ immune cells among CD45^+^ cells was markedly higher in the 70 mg/kg RTS/PH (81.64% ± 2.295%, *n* = 5) groups than that in the control group (67.76% ± 8.054%, *n* = 5), while the 0 mg/kg RTS/PH (72.41% ± 2.859%, *n* = 5) and 35 mg/kg RTS/PH (77.06% ± 10.42%, *n* = 5) showed no significant difference (Figure 2C). Furthermore, the proportion of CD11b^+^Ly6G^+^ MDSCs among CD45^+^ immune cells was significantly higher in the 70 mg/kg RTS/PH group (35.10% ± 2.877%, *n* = 5) than that in the control group (23.90% ± 6.874%, *n* = 5), with no significant differences in 0 mg/kg RTS/PH (20.16% ± 3.389%, *n* = 5) and 35 mg/kg RTS/PH (22.00% ± 6.345%, *n* = 5) groups (Figure 2D). These results indicate that suppression of hepatocyte proliferation and HPC activation enhances CD11b^+^Ly6G^+^ MDSC infiltration.

### Liver recovery was impaired by day 14 post-PH when hepatocyte proliferation was suppressed by RTS

**Figure 3.**
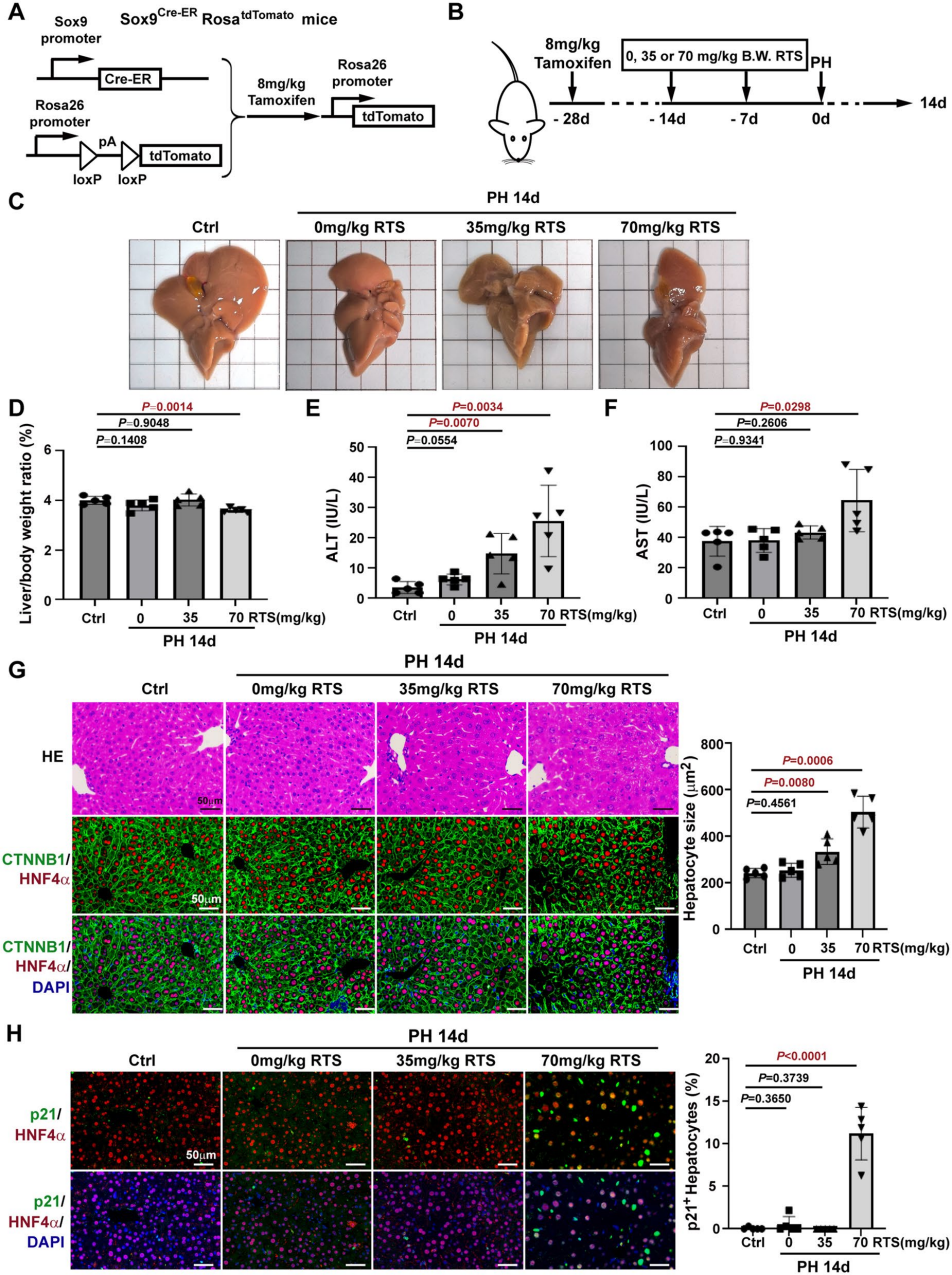
**RTS (70 mg/kg) impaired liver recovery by day 14 post-PH.** (A) Sox9^Cre-ER^Rosa^tdTomato^ mice were given a single dose of tamoxifen to activate tdTomato expression in Sox9 expressing cells. (B) Experimental design to determine the effects of different dose of RTS on Sox9^Cre-ER^Rosa^tdTomato^ mice by day 14 post-PH. (C) The representative macroscopic view of livers from control, 0 mg/kg, 35 mg/kg, or 70 mg/kg RTS/PH mice by day 14 post-PH. (D) The liver-to-body weight ratio was markedly lower in 70 mg/kg RS/PH mice compared to controls by day 14 post-PH. (E) Seum ALT levels in the control, 0 mg/kg, 35 mg/kg, or 70 mg/kg RTS/PH mice. (F) Seum AST levels in the control, 0 mg/kg, 35 mg/kg, or 70 mg/kg RTS/PH mice. (G) H&E and β-Catenin/HNF4α immunostaining showed significant cell size increase in 35 mg/kg and 70 mg/kg RTS/PH mice compared to controls by day 14 post-PH. (H) Immunostaining of p21 and HNF4α revealed about 12% p21-positive hepatocytes in the 70 mg/kg RTS/PH mice by day 14 post-PH.

Sox9^Cre-ER^Rosa^tdTomato^ mice were used to trace HPCs and determine their contribution to liver recovery (Figure 3A). The Sox9^+^ HPCs were labeled with a single dose (8 mg/kg) of tamoxifen followed by a 2-week washout (Figure 3A), which was lower than the 32 mg/kg reported previously [23], to avoid ectopic Sox9 expression in hepatocytes induced by high tamoxifen doses [24]. The mice received 35 or 70 mg/kg RTS twice before PH (Figure 3B). Two mice in the 70 mg/kg RTS group died within 24 h post-PH. The remnant livers of surviving mice appeared enlarged by day 14 (Figure 3C). The liver-to-body weight ratio remained significantly lower in 70 mg/kg RTS/PH mice (3.610% ± 0.09121%, *n* = 5) than that in controls (4.005% ± 0.1611%, *n* = 5). In contrast, the liver-to-body weight ratios in the 0 mg/kg RTS/PH (3.807% ± 0.2175%, *n* = 5) and 35 mg/kg RTS/PH (4.021% ± 0.2416%, *n* = 5) groups were similar to that in the control group (Figure 3D), confirming impaired liver regeneration after 70 mg/kg RTS/PH. Furthermore, the mean ALT levels were all lower than 30 IU/mL in each group (Figure 3E), while AST levels were markedly increased in 70 mg/kg RTS/PH (64.50 ± 20.54, *n* = 5) groups than that in the control group (37.62 ± 9.835, *n* = 5) (Figure 3F). Additionally, H&E staining and double immunofluorescence staining for HNF4α and β-Catenin revealed that the hepatocyte size was significantly increased in 35 mg/kg RTS/PH (335.3 ± 55.26 μm^2^, *n* = 5) and 70 mg/kg RTS/PH mice (505.3 ± 68.29 μm^2^, *n* = 5) compared to that in control mice (243.0 ± 20.06 μm^2^, *n* = 5) (Figure 3G). HPCs differentiate into hepatocytes when hepatocytes express senescence marker p21 [25]. Liver sections co-stained for p21 and HNF4α revealed only a few p21^+^ hepatocytes in the control, 0 mg/kg RTS/PH, or 35 mg/kg RTS/PH groups, whereas approximately 12% hepatocytes were positive for p21 in the 70 mg/kg RTS/PH group by day 14 (Figure 3H). These data suggested that RTS impaired liver recovery and induced hepatocyte hypertrophy and senescence by day 14 post-PH.

### Minimal HPC contribution to liver regeneration in 70 mg/kg RS/PH mice

**Figure 4.**
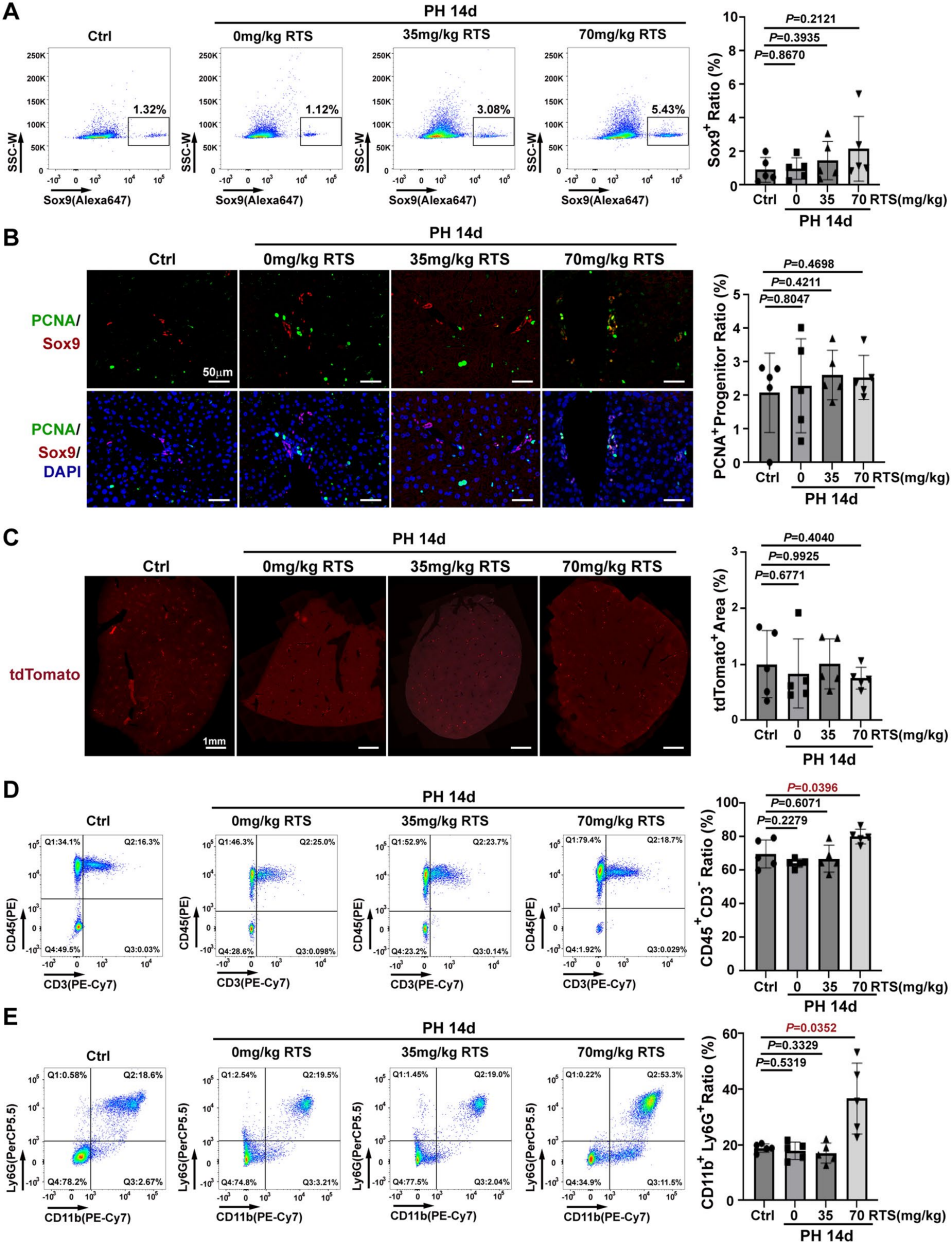
**No significant expansion of HPCs in RTS (70 mg/kg)/PH mice by day 14 post-PH.** (A) No significant changes in the proportion of Sox9^+^ HPCs in 70 mg/kg RS/PH mice compared to controls. (B) Few PCNA^+^ HPCs in 70 mg/kg RTS/PH mice compared to controls. (C) Representative whole-slide scanning of liver sections revealed minimal difference of tdTomato-positive regenerative foci between 70 mg/kg RTS/PH mice and controls by day 14 post-PH. (D) The proportion of CD45^+^CD3^–^ immune cells increased in 70 mg/kg RTS/PH group compared to controls by day 14 post-PH. (E) The proportion of CD11b^+^Ly6G^+^ MDSCs were higher in 70 mg/kg RTS/PH mice compared to controls by day 14 post-PH.

In the progenitor cell analysis, flow cytometry showed no significant difference in the proportion of Sox9^+^ HPCs among the NPCs in the 0 mg/kg RTS/PH (0.9840 ± 0.6487, *n* = 5), 35 mg/kg RTS/PH (1.458 ± 1.147, *n* = 5), and 70 mg/kg RS/PH (2.160 ± 1.928, *n* = 5) groups and controls (0.9080 ± 0.7379, *n* = 5) by day 14 (Figure 4A). Similarly, double immunofluorescence staining for PCNA and Sox9 revealed no significant difference in PCNA^+^ progenitor cell proportion among the 0 mg/kg RTS/PH (2.288 ± 1.401, *n* = 5), 35 mg/kg RS/PH (2.608 ± 0.7388, *n* = 5), 70 mg/kg RS/PH (2.539 ± 0.6598, *n* = 5) and control groups (2.079 ± 1.186, *n* = 5) (Figure 4B), indicating minimal HPC expansion. Additionally, immunostaining for the lineage tracing marker tdTomato and whole-slide scanning revealed no significant increase in tdTomato-positive regenerative foci in any PH group compared to that in the control group by day 14, including the 35 mg/kg RTS/PH mice, that showed HPC activation by day 2 post PH (Figure 4C). Thus, similar to hepatocyte-proliferating conditions (0 and 35 mg/kg RTS/PH), HPCs showed minimal contribution to liver recovery, even in RTS-induced hepatocyte senescence conditions (70 mg/kg RTS/PH).

Subsequently, liver immune cell analysis showed significantly higher proportions of CD45^+^CD3^－^ immune cells (80.19% ± 4.354% versus 69.77% ± 8.427%, Figure 4D) and CD11b^+^Ly6G^+^ MDSCs (36.62% ± 12.74% versus 18.88% ± 1.496%, Figure 4E) in 70 mg/kg RTS/PH mice (*n* = 5) than that in the control group (*n* = 5). In contrast, both ratios in the 0 and 35 mg/kg RTS/PH groups were similar to that in the control group by day 14 post-PH (Figure 4D and E), suggesting liver regeneration failure accompanied with CD11b^+^Ly6G^+^ MDSC infiltration.

### RTS induces progenitor cell senescence by enhancing ROS production

**Figure 5.**
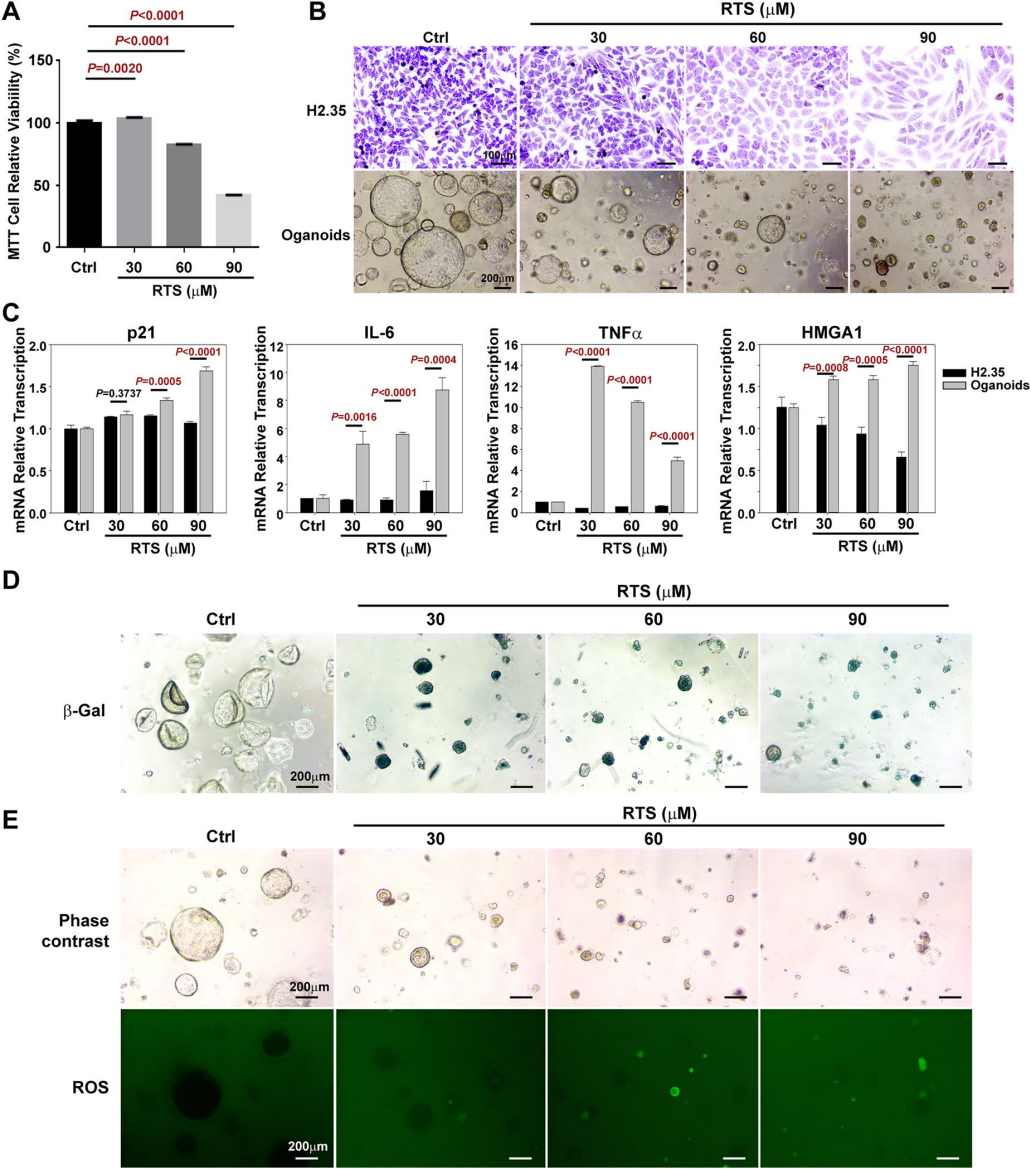
**RTS induced HPC senescence** *via* **enhancing ROS production** *in vitro*. (A) MTT analyses showed RTS dose-dependently reduced the viability of *H2.35* cells. (B) Giemsa staining of *H2.35* showed RTS dose-dependently suppressed proliferation and induced enlargement of cell size, and phase contrast microscope pictures showed the dose-dependent growth inhibitory effects of RTS on mouse liver organoids. (C) RTS did not increase the mRNA levels of p21, IL-6, TNFα and HMGA1 in *H2.35* cells, but markedly enhanced these senescence mRNA transcriptions in liver organoids. (D) RTS treatment enhanced the β-gal activity of liver organoids. (E) RTS exhibited a dose-dependent increase in ROS levels within liver organoids.

The hepatocyte cell line H2.35 and HPC-enriched liver organoids were treated with different doses of RTS *in vitro* to assess why HPCs failed to expand and differentiate in 70 mg/kg RTS/PH mice. RTS inhibited H2.35 viability in the MTT assay in a dose-dependent manner (Figure 5A) and induced cell enlargement, as evidence by Giemsa staining (Figure 5B). Similarly, RTS inhibited organoid growth in a dose-dependent manner (Figure 5B). qRT-PCR results showed that RTS markedly enhanced mRNA levels of the senescence marker p21 and senescence-associated secretory phenotype (SASP) factors (IL-6, TNFα, HMGA1) in organoids at concentrations of 60 μM and 90 μM, with effects significantly greater than that in H2.35 cells treated with equivalent doses of RTS (Figure 5C). Additionally, β-Gal staining demonstrated that RTS induced senescence in organoids at concentrations as low as 30 μM (Figure 5D), which did not significantly suppress H2.35 proliferation. ‌Measurement of ROS, a major regulator of cell senescence, revealed that RTS increased ROS levels in organoids at concentrations of 60 μM and 90 μM (Figure 5E).

**Figure 6.**
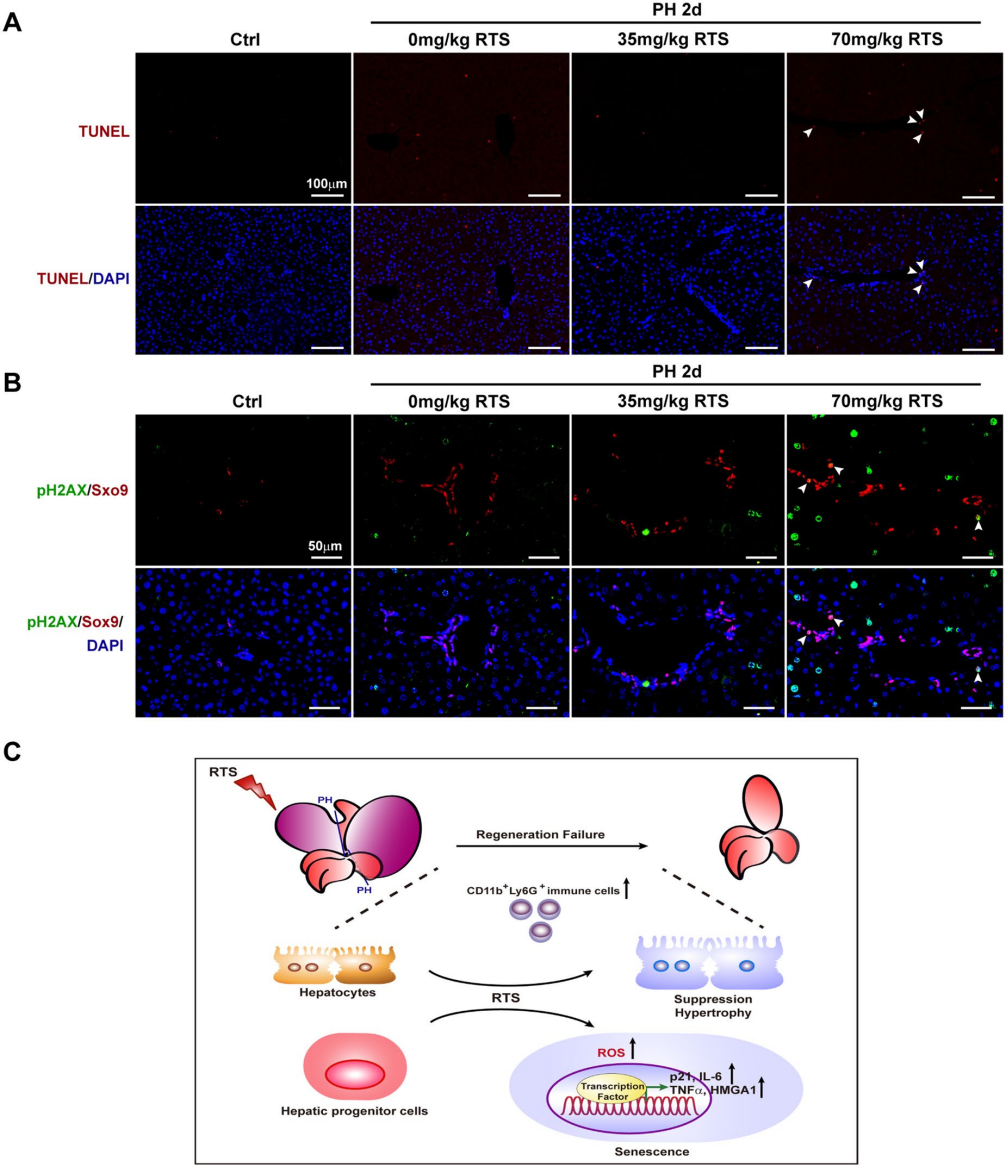
**RTS induced HPC senescence of HPC** *in vivo*. (A) Few TUNEL-positive cells could be found in the control, 0 mg/kg, and 35 mg/kg RTS/PH mice, whereas some TUNEL-positive cholangiocytes (arrow head) could be detected in 70 mg/kg RS/PH mice at 2-day post PH. (B) Few Sox9/phosphor-Histone H2A.X double positive cells could be found in the control, 0 mg/kg, and 35 mg/kg RTS/PH mice, whereas some of these cells (arrow head) could be detected in 70 mg/kg RS/PH mice at 2-day post PH. (C) Schematic representation of the mechanism in RTS-induced regeneration failure after PH. RTS induces HPC senescence *via* ROS when hepatocyte proliferation is totally suppressed. Hepatocyte hypertrophy and CD11b^+^Ly6G^+^ MDSC infiltration are involved in RTS-induced regeneration failure process.

Given the high mortality (2/7 prior to analysis) and severe hepatotoxicity observed in the 70 mg/kg RTS/PH mice, we performed TUNEL staining to determine whether RTS-induced regeneration failure was due to overwhelming cell death or impaired proliferation. As shown in Figure 6A, TUNEL-positive staining was minimal in control, 0 mg/kg, and 35 mg/kg RTS/PH mice, whereas specific cholangiocytes in the 70 mg/kg group showed positivity at 2 days post-PH, suggesting that widespread cell death was not the primary cause of RTS-induced regeneration failure. As RTS induced senescence in HPCs *in vitro*, we sought to validate this finding *in vivo* using double immunofluorescence staining for Sox9 and the senescence marker phospho-Histone H2A.X (Ser139). As expected, few Sox9/phospho-Histone H2A.X double-positive HPCs could be found in control and lower-dose groups but these cells were detectable in the 70 mg/kg RTS/PH mice at 2 days post-PH (Figure 6B), confirming the RTS-induced senescence in HPCs. In summary, RTS induced HPC senescence by enhancing ROS generation, resulting in minimal contribution of HPC to liver regeneration when hepatocyte proliferation was completely suppressed by RTS (Figure 6C).

## Discussion

This study provided new insights into liver regeneration failure post-PH under RTS conditions that suppress hepatocyte proliferation. We found that RTS-induced HPC senescence accounts for liver regeneration failure when hepatocyte proliferation is completely suppressed by RTS after PH. Additionally, RTS targets HPCs in the liver, in addition to hepatocytes and sinusoidal endothelial cells, and induces HPC senescence *via* ROS generation. Furthermore, RTS-induced regeneration failure was accompanied by hepatocyte hypertrophy and CD11b^+^Ly6G^+^ MDSC infiltration, suggesting that hepatocyte compensation and immune cell participation are involved in the regeneration failure process.

Two major cell populations in adult rodent livers are responsible for liver weight restoration: hepatocyte duplication for acute injuries and HPC activation/differentiation after chronic injuries or when hepatocyte proliferation is compromised [6,26–28]. Our study found that HPCs were activated with minimal hepatocyte differentiation when hepatocyte proliferation was partially suppressed by 35 mg/kg RTS, which is consistent with the results of previous studies in which hepatocyte duplication mainly contributed to liver recovery after PH [3,4]. In contrast to the rat AAF/PH HPC activation model, AAF/PH [7–10], our study revealed that the mouse RTS/PH model is a regeneration failure model without HPC expansion. Minimal hepatocyte differentiation was observed in HPCs when hepatocyte proliferation was suppressed and 12% of hepatocytes were in the senescent state, which is different from hepatocyte differentiation of HPC in the induced expression of E3 ubiquitin ligase Mdm2 mice with nearly all hepatocytes in senescence [6]. This divergence may be due to the RTS-induced senescence of HPC, whereas HPCs were not injured in the induced expression of E3 ubiquitin ligase in Mdm2 mice. Thus, our results indicate that injury to HPCs leads to liver regeneration failure when hepatocyte duplication is suppressed.

RTS is a well-known pyrrolizidine alkaloid that is widely distributed in plants, and is often obtained *via* consumption of herbs, tea, or contaminated food [14–16]. RTS damages hepatocytes [29,30] and impairs sinusoidal endothelial cells [31]. In this study, we found that HPCs are another cell type targeted by RTS, which blocks HPC activation when hepatocyte proliferation is compromised. Mechanistically, RTS increases ROS levels in HPCs, similar to RTS-induced oxidative stress in HepaRG cells [32] and rat hepatocytes [33], thereby inducing the senescence of HPCs. Thus, the results of this study identified the liver cell types experiencing cellular senescence [34] and deepen our understanding of RTS-induced liver injury.

Hepatocyte hypertrophy serves as a crucial compensatory mechanism to maintain liver function and prevent liver failure when hepatocyte proliferation is impaired in various genetic models (e.g. Skp2 KO, liver-specific STAT3 KO, liver-specific Pdk1 KO, and Pdk1/STAT3 double KO) [35–37]. Hepatocyte size increases after 70% PH, and this enlargement is sustained until 14-day post PH even after liver weight recovery [38,39]. In this study, we did not observe significant hepatocyte enlargement after 50% PH but observed marked hepatocyte enlargement when hepatocyte proliferation was partially or completely suppressed. Similar to a previous study, the enlarged hepatocyte size was sustained up to 14-day post PH after liver weight recovery in mice was partially suppressed by RTS. Moreover, complete suppression of hepatocyte proliferation leads to larger hepatocytes than does partial suppression, representing functional adaptation by surviving hepatocytes when HPCs are senescent and regeneration fails.

Specific immune cell subsets such as MARCO^+^ macrophages [40] and bone marrow-derived CD11b^+^ Kupffer cells [41], promote regeneration post-PH. In this study, we found that the ratio of CD45^+^CD3^－^ immune cells was elevated in the 70 mg/kg RTS/PH groups by day 2 and 14 post-PH accompanied with regeneration failure. Specifically, the increased number of CD11b^+^Ly6G^+^ MDSCs in 70 mg/kg RTS/PH mice by day 2 and 14 post-PH, which was not observed in the control, 0 mg/kg, and 35 mg/kg RTS/PH mice, suggesting that CD11b^+^Ly6G^+^ MDSCs are an essential immune cell subtype involved in the RTS-impaired regeneration process, which requires further exploration.

## Conclusions

HPCs contribute minimally to liver regeneration when hepatocyte proliferation is completely arrested by RTS, leading to liver recovery failure. This regeneration-failure process is accompanied by compensatory hepatocyte hypertrophy and CD11b^+^Ly6G^+^ MDSC infiltration. These findings provide a model for studying the mechanisms of regeneration failure and exploring new therapeutic strategies to rescue regeneration failure after resection due to HPC senescence.

## Supplementary Material

Supplemental Material

Supplemental Material

## Data Availability

Data and materials used in this study are available from the corresponding author upon request.

## Open scholarship

This article has earned the Center for Open Science badges for Open Materials. The materials are openly accessible at https://doi.org/10.5281/zenodo.18586474.
