# Supplementary material for: Retrorsine impairs liver regeneration by inducing progenitor cell-senescence via ROS after partial hepatectomy
Source: Ann Med. 2026 Mar 3;58(1):2635821. doi: 10.1080/07853890.2026.2635821 (PMC12961708; doi:10.1080/07853890.2026.2635821)
Supplement: Supplemental Material [file IANN_A_2635821_SM1604.docx]

**Supplementary Table 2. List of primers used in this study.**

| Name | Primer sequence | Gene symbol |
| --- | --- | --- |
| GAPDH | F: AGGTCGGTGTGAACGGATTTG | NM_001289726.2 |
|  | R: TGTAGACCATGTAGTTGAGGTCA |  |
| HMGA1 | F: GGTCGGGAGTCAGAAAGAGC | NM_001166476.1 |
|  | R: ATTCTTGCTTCCCTTTGGTCG |  |
| IL-6 | F: TAGTCCTTCCTACCCCAATTTCC | NM_031168.2 |
|  | R: TTGGTCCTTAGCCACTCCTTC |  |
| p21 | F: CCTGGTGATGTCCGACCTG | NM_007669.5 |
|  | R: CCATGAGCGCATCGCAATC |  |
| TNFα | F: CCTGTAGCCCACGTCGTAG | NM_013693.3 |
|  | R: GGGAGTAGACAAGGTACAACCC |  |
