# Supplementary material for: Retrorsine impairs liver regeneration by inducing progenitor cell-senescence via ROS after partial hepatectomy
Source: Ann Med. 2026 Mar 3;58(1):2635821. doi: 10.1080/07853890.2026.2635821 (PMC12961708; doi:10.1080/07853890.2026.2635821)
Supplement: Supplemental Material [file IANN_A_2635821_SM1603.docx]

**Supplementary Table 1: List of antibodies used in this study.**

| Antibodies | Company | Catalog Number | Clone number |
| --- | --- | --- | --- |
| BV421 anti-mouse CD45 Antibody | Biolegend | 103134 | [30-F11](https://www.biolegend.com/en-us/search-results?Clone=30-F11) |
| PE/Cyanine7 anti-mouse CD3 Antibody | Biolegend | 100220 | 17A2 |
| PE/Cyanine7 anti-mouse CD11b Antibody | Biolegend | 101216 | M1/70 |
| PerCP/Cyanine5.5 anti-mouse Ly-6G Antibody | Biolegend | 127616 | 1A8 |
| PE anti-mouse CD45 Antibody | Biolegend | 147712 | I3/2.3 |
| Alexa Fluor 647 Mouse Anti-Sox9 | BD Biosciences | 565493 | T32-668 |
| Anti-HNF-4-alpha | Abcam | ab41898 | K9218 |
| Sox9 Rabbit mAb | Cell Signaling Technology | #82630 | D8G8H |
| PCNA mAb | Cell Signaling Technology | #2586 | PC10 |
| tdTomato antibody | Biorbyt | orb182397 |  |
| HNF4αRabbit mAb | Cell Signaling Technology | #3113 | C11F12 |
| p21 polyclone antibody | ProteinTech | 10355-1-AP |  |
| β-Catenin Rabbit PolyAb | ProteinTech | 51067-2-AP |  |
| phospho-Histone H2A.X mAb | Cell Signaling Technology | #80312 | D7T2V |
| Donkey anti-Mouse IgG (H+L) Highly Cross-Adsorbed Secondary Antibody, Alexa Fluor™ 488 | Molecular Probes | A21202 |  |
| Donkey anti-Rabbit IgG (H+L) Highly Cross-Adsorbed Secondary Antibody, Alexa Fluor™ 488 | Molecular Probes | A21206 |  |
| Donkey anti-Goat IgG (H+L) Cross-Adsorbed Secondary Antibody, Alexa Fluor™ 555 | Molecular Probes | A21432 |  |
| Donkey anti-Mouse IgG (H+L) Highly Cross-Adsorbed Secondary Antibody, Alexa Fluor™ 555 | Molecular Probes | A31570 |  |
| Donkey anti-Rabbit IgG (H+L) Highly Cross-Adsorbed Secondary Antibody, Alexa Fluor™ 555 | Molecular Probes | A31572 |  |
